# Novel mutations of PKD genes in Chinese patients suffering from autosomal dominant polycystic kidney disease and seeking assisted reproduction

**DOI:** 10.1186/s12881-018-0693-7

**Published:** 2018-10-17

**Authors:** Wen-Bin He, Wen-Juan Xiao, Yue-Qiu Tan, Xiao-Meng Zhao, Wen Li, Qian-Jun Zhang, Chang-Gao Zhong, Xiu-Rong Li, Liang Hu, Guang-Xiu Lu, Ge Lin, Juan Du

**Affiliations:** 1 Institute of Reproductive and Stem Cell Engineering, School of Basic Medical Science, Central South University, Changsha, Hunan 410078 People’s Republic of China; 2 Reproductive and Genetic Hospital of CITIC-Xiangya, Changsha, Hunan 410078 People’s Republic of China

**Keywords:** Autosomal dominant polycystic kidney disease, *PKD1* gene, *PKD2* gene, *GANAB* gene, Novel mutation, Male infertility

## Abstract

**Background:**

Autosomal dominant polycystic kidney disease (ADPKD), the commonest inherited kidney disease, is generally caused by heterozygous mutations in *PKD1*, *PKD2,* or *GANAB* (*PKD3*).

**Methods:**

We performed mutational analyses of PKD genes to identify causative mutations. A set of 90 unrelated families with ADPKD were subjected to mutational analyses of PKD genes. Genes were analysed using long-range PCR (LR-PCR), direct PCR sequencing, followed by multiplex ligation-dependent probe amplification (MLPA) or screening of *GANAB* for some patients. Semen quality was assessed for 46 male patients, and the correlation between mutations and male infertility was analysed.

**Results:**

A total of 76 mutations, including 38 novel mutations, were identified in 77 families, comprising 72 mutations in *PKD1* and 4 in *PKD2*, with a positive detection rate of 85.6%. No pathogenic mutations of *GANAB* were detected. Thirty-seven patients had low semen quality and were likely to be infertile. No association was detected between *PKD1* mutation type and semen quality. However, male patients carrying a pathogenic mutation in the Ig-like repeat domain of *PKD1* had a high risk of infertility.

**Conclusion:**

Our study identified a group of novel mutations in PKD genes, which enrich the PKD mutation spectrum and might help clinicians to make precise diagnoses, thereby allowing better family planning and genetic counselling. Men with ADPKD accompanied by infertility should consider intracytoplasmic sperm injection combined with preimplantation genetic diagnosis to achieve paternity and obtain healthy progeny.

**Electronic supplementary material:**

The online version of this article (10.1186/s12881-018-0693-7) contains supplementary material, which is available to authorized users.

## Background

Autosomal dominant polycystic kidney disease (ADPKD) is the commonest inherited kidney disease, with an estimated incidence of 1:400 to 1:1000; it accounts for 7–10% of all patients on renal replacement therapy worldwide [1]. It is characterised by the development of renal cysts, hypertension, and extrarenal cysts, and results in end-stage renal disease (ESRD) [2]. In addition, male patients with ADPKD usually show infertility resulting from cystic dilatation of the seminal vesicles [3].

ADPKD is an autosomal dominant inherited disorder resulting from heterozygous mutations in three genes: *PKD1*, *PKD2,* and *GANAB*. Mutations of the first two genes (*PKD1* and *PKD2*) account for 80–85% and 15–20% of resolved cases, respectively [4, 5]. As of January 31, 2018, more than 2000 mutations (2323 in *PKD1* and 278 in *PKD2*) had been described in the Autosomal Dominant Polycystic Kidney Disease Mutation Database (PKDB; http://pkdb.mayo.edu/). Recently, two studies reported the association of the third ADPKD gene *GANAB*, or *PKD3* [6, 7], which accounts for ~ 0.3% of the total cases of ADPKD [7].

In the present study, we performed mutational screening of *PKD1*, *PKD2,* and *GANAB* using long-range PCR (LR-PCR) and direct sequencing, as well as multiplex ligation-dependent probe amplification (MLPA) in 90 unrelated Chinese families with ADPKD. A total of 76 likely pathogenic or pathogenic mutations were identified in 77 families, including 38 novel mutations in PKD genes. These mutation data will contribute to improvement of diagnostics and genetic counselling in a clinical setting. In addition, this study highlights the correlation between men with ADPKD and infertility.

## Methods

### Study subjects

A total of 90 unrelated families were recruited from the Reproductive and Genetic Hospital of CITIC-Xiangya in China from October 2012 to October 2017, including 72 patients with a positive family history of ADPKD. These patients either sought genetic counselling to avoid delivering a baby with ADPKD due to a positive family history, or sought treatment at our hospital for infertility and were diagnosed with ADPKD based on ultrasound examination before undergoing assisted reproductive technology treatment. All diagnoses were confirmed by ultrasound examination according to previously described criteria: (1) the presence of at least three (unilateral or bilateral) renal cysts in individuals aged 15 to 39 years, or (2) the presence of at least two cysts in each kidney in individuals aged 40 to 59 years, or (3) the presence of four or more cysts in each kidney in individuals aged 60 years and above [8].

All individuals signed a written informed consent form, and blood samples were obtained from all probands and their family members when possible. The study was approved by the Ethics Committee of the Reproductive and Genetic Hospital of CITIC-Xiangya.

### Semen analysis and assisted reproductive therapies

Among the 90 unrelated ADPKD probands, 61 were male and 46 provided semen specimens for analysis. Specimens were collected by means of masturbation into a sterile container after 2–7 days of abstinence. All specimens were assessed according to the World Health Organization (WHO) 2010 recommendations [9]. Briefly, within 1 h of ejaculation, the samples were liquefied and analysed for semen volume, sperm concentration, round cells, normal morphology, and sperm motility (defined as WHO motility grades A, B, C, and D, where grade A indicates fast progressive sperm; B, slow progressive sperm; C, nonprogressive sperm; and D, immotile sperm).

All patients providing semen specimens for analysis had a normal 46, XY karyotype, and no Y chromosome abnormalities were detected by microdeletion detection. Other causes of infertility, such as drugs and exposure to toxic substances, were excluded. Physical examination of these male patients showed normal results, including height, weight, hair distribution, mental state, and external genital organs.

Most of individuals who provided semen specimens for analysis have chosen different approaches to conceive offspring, including natural pregnancy, in vitro fertilization (IVF), intracytoplasmic sperm injection (ICSI), and ICSI combined preimplantation genetic diagnosis (PGD).

### Mutation analysis of *PKD1*, *PKD2,* and *GANAB*

Genomic DNA (gDNA) was extracted from peripheral blood samples using a QIAamp® DNA blood midi kit (QIAGEN, Hilden, Germany) according to the manufacturer’s protocol. All patients were subjected to mutation screening of *PKD1* and *PKD2* using Sanger sequencing, followed by multiplex ligation-dependent probe amplification analysis (MLPA) to detect copy number variation in *PKD1* and *PKD2* in patients lacking definitely pathogenic point mutations in *PKD1* or *PKD2*. Subsequent screening of *GANAB* was carried out in patients for whom no causative genetic aetiology in *PKD1* and *PKD2* had been identified.

For exons 1–34 of *PKD1*, LR-PCR followed by nested PCR was performed with *PKD1*-specific primers, as previously described [10–12], and exons 35–46 of *PKD1* were directly amplified from gDNA by PCR. All exons of *PKD2* and *GANAB,* including the adjacent 30–60 bp intron sequence, were amplified from gDNA by PCR. The primers for amplification of *PKD1* were previously described, with minor modifications [12, 13]; specific primers for *PKD2* and *GANBA* were designed using Primer 3 online (http://primer3.ut.ee) according to reference sequences. The primers and conditions for PCR reactions are provided in Additional file 1: Table S1, Additional file 2: Table S2, Additional file 3: Table S3, Additional file 4: Table S4. If a variant was identified as a putative disease-causative mutation, then mutation site screening of family members was implemented. DNA samples from all patients were screened by bidirectional sequencing on an Applied Biosystems 3130XL genetic analyser (Applied Biosystems, Foster City, CA, USA).

Copy number variation analysis of *PKD1* and *PKD2* was performed by MLPA with a SALSA MLPA probemix P351-C1/P352-D1 *PKD1-PKD2* kit (MRC-Holland, Amsterdam, the Netherlands) according to the manufacturer’s instructions [14]. This kit contains probes for 36 of the 46 exons of *PKD1* and 17 probes for *PKD2* exons, covering all PKD2 exons except exon 13 (two probes each for PKD2 exons 1, 2, and 6). The results of MLPA analysis were scanned on an Applied Biosystems 3130XL genetic analyser (Applied Biosystems, Foster City, CA, USA). The raw data were analysed using the Coffalyser MLPA analysis tool (MRC-Holland, Amsterdam, the Netherlands).

### Evaluation of the pathogenicity of variations

The PKDB (http://pkdb.mayo.edu), the Human Gene Mutation Database (HGMD; http://www.hgmd.cf.ac.uk/ac/index.php), Exome Aggregation Consortium (EXAC; http://exac.broadinstitute.org), and the gnomAD database (http://gnomad.broadinstitute.org) were searched for previously reported variations. Frameshift variations, typical splicing, nonsense, and in-frame changes of two or more amino acids were defined as definitely pathogenic mutations [2, 15]. A novel mutation was defined as one that had not been described in PKDB or HGMD, or reported in ADPKD patients. The potential pathogenicity of all identified missense variants, indicated by a frequency below 1% in the Asian population of the Exac and gnomAD databases, was evaluated by pedigree analysis and in silico analysis using three different tools: SIFT (http://sift.bii.a-star.edu.sg/), Polyphen-2 (http://genetics.bwh.harvard.edu/pph2), and MutationTaster (http://www.mutationtaster.org/). All variants were classified into five categories: ‘pathogenic’, ‘likely pathogenic’, ‘uncertain significance’, ‘likely benign’, and ‘benign’, according to the American College of Medical Genetics and Genomics (ACMG) standards and guidelines for the interpretation of variations [16].

## Results

### Mutation analysis of *PKD1, PKD2,* and *GANAB*

We performed mutation screening of *PKD1*and *PKD2* for 90 probands using Sanger sequencing and MLPA. The 33 probands for whom no definitely pathogenic mutations were detected in *PKD*1 and *PKD2* were subjected to screening of *GANAB*. A total of 93 variations were identified in this study, comprising 84, 4, and 5 variations in *PKD1*, *PKD2,* and GANAB, respectively (Tables 1 and 2). Among these variations, 51 are novel and have not been described in the ADPKD Mutation Database or HGMD, or been reported in ADPKD patients.

**Table 1:** Defnitely pathogenic mutations in PKD1 and PKD2 identified in this study

cDNA change	Exon/ intron	Amino acid change	Mutation Type	Family No.	Family history	Known/Novel
*PKD1*						
c.74dupG	1	p.Gly25Glyfs*89	Frameshift	29	Yes	Novel
c.106_107insT	1	p.Pro36Leufs*78	Frameshift	12	Yes	Novel
c.467_487del21	4	p.Ala156_Ala162del	In-frame deletion	49	Yes	Novel
c.856_862delTCTGGCC	5	p.Ser286Serfs*2	Frameshift	30	Yes	Known
c.1198C > T	5	p.Arg400*	Nonsense	17	Yes	Known
c.1297C > T	6	p.Gln433*	Nonsense	48	NA	Known
c.2050A > T	10	p.Arg684*	Nonsense	47	Yes	Novel
c.2659delT	11	p.Trp887Glyfs*11	Frameshift	50	Yes	Known
c.2670 + 1G > A	IVS14	-	Splice	19	Yes	Novel
c.4177C > T	15	p.Gln1393*	Nonsense	51	Yes	Novel
c.4447C > T	15	p.Gln1483*	Nonsense	13	Yes	Known
				14	Yes	
c.4551C > A	15	p.Tyr1517*	Nonsense	39	Yes	Novel
c.4609G > T	15	p.Glu1537*	Nonsense	31	Yes	Known
c.4846G > T	15	p.Glu1616*	Nonsense	37	Yes	Novel
c.4957C > T	15	p.Gln1653*	Nonsense	16	Yes	Known
c.5014_5015delAG	15	p.Arg1672Glyfs*98	Frameshift	53	Yes	Known
c.5120G > A	15	p.Trp1707*	Nonsense	26	Yes	Known
c.5637C > G	15	p.Tyr1879*	Nonsense	20	Yes	Novel
c.6115C > T	15	p.Gln2039*	Nonsense	55	Yes	Known
c.6199C > T	15	p.Gln2067*	Nonsense	34	Yes	Known
c.6804delG	15	p.Trp2268Cysfs*46	Frameshift	63	No	Novel
c.6813_6814delAC	15	p.Arg2272Glyfs*147	Frameshift	7	Yes	Known
c.6945_6946insT	16	p.Gly2316Trpfs*104	Frameshift	1	Yes	Novel
c.7126C > T	17	p.Gln2376*	Nonsense	45	Yes	Known
c.7863 + 1G > C	IVS20	-	Splice	36	Yes	Novel
c.7863 + 2 T > G	IVS20	-	Splice	11	Yes	Novel
c.7915C > T	21	p.Arg2639*	Nonsense	54	Yes	Known
c.7973_7974delTG	21	p.Val2658Glyfs*2	Frameshift	9	Yes	Known
c.8338G > T	23	p.Glu2780*	Nonsense	27	Yes	Known
c.9666_9667delGA	28	p.Glu3222Aspfs*30	Frameshift	32	Yes	Novel
c.10050 + 1G > A	IVS30	-	Splice	44	Yes	Known
c.10220 + 2 T > C	IVS32	-	Splice	3	Yes	Known
c.10397C > G	34	p.Ser3466*	Nonsense	6	Yes	Novel
c.10524_10525delAG	35	p.Glu3509Aspfs*117	Frameshift	2	NA	Novel
c.10710_10715delGGCTGT	36	p.3571_3572del2	In-frame deletion	40	Yes	Known
c.10724G > A	36	p.Try3575*	Nonsense	38	NA	Novel
c.10896_10897delGA	37	p.Ser3633Profs*88	Frameshift	5	No	Novel
c.11240delC	39	p.Pro3747Hisfs*79	Frameshift	33	Yes	Novel
c.11269 + 1G > A	IVS39	-	Splice	10	Yes	Novel
c.11311_11312insGTGCT	40	p.Ser3771Cysfs*57	Frameshift	41	NA	Novel
c.11512C > T	41	p.Gln3838*	Nonsense	15	Yes	Known
c.11538-2A > G	IVS41	-	Splice	18	Yes	Known
c.11617_11637del21	42	p.3873_3879del7	In-frame deletion	4	Yes	Novel
c.11699_11700ins10	42	p.Leu3901Alafs*63	Frameshift	22	Yes	Novel
c.11830_11838dup	43	p.Leu3944_Ala3946dup	In-frame duplication	52	Yes	Novel
c.12101delT	44	p.Val4034Glyfs*5	Frameshift	25	No	Novel
c.12139-2A > T	IVS44	-	Splice	24	Yes	Novel
c.12391G > T	45	p.Glu4131*	Nonsense	43	Yes	Known
c.12570_12571insCTCC	46	p.Ser4190Serfs*21	Frameshift	28	Yes	Novel
c.12682C > T	46	p.Arg4228*	Nonsense	21	Yes	Known
				23	Yes	
c.12712C > T	46	p.Gln4238*	Nonsense	46	Yes	Known
EX31-33del	31–33	-	Large deletion	72	No	Novel
*PKD2*						
c.973C > T	4	p.Arg325*	Nonsense	8	Yes	Known
c.1094 + 3_1094 + 6delAAGT	IVS4	-	Splice	35	Yes	Known
c.2159dupA	11	p.Asn720Lysfs*5	Frameshift	42	Yes	Known

*NA* not available; *translation termination codon. Novel mutation defined as one that had not been described in PKDB, HGMD, or reported in ADPKD patients

**Table 2:** Evaluation of the pathogenic potential of PKD genes missense variants

cDNA change	Exon	Amino acid change	Co-occurence	SIFT	PolyPhen-2	Mutation Taster	Family No.	Family history	Segregation	Known/Novel	Classification
*PKD1*											
c.1385G>T	6	p.Arg462Met		NT	PRD	D	77	Yes	Yes	Novel	LP
c.2039A > T	10	p.Tyr680Phe	p.Tyr1879*	NT	POD	P	20	Yes	Yes	Known	LB
c.2180 T>C	11	p.Leu727Pro		NT	PRD	D	69	Yes	Yes	Known	LP
c.2897G>C	12	p.Arg966Pro		NT	PRD	D	73	Yes	Yes	Novel	LP
c.3548C > G	15	p.Ser1183Trp	p.Gln1653*	NT	B	P	16	Yes	Yes	Novel	LB
c.3613G>C	15	p.Asp1205His		NT	POD	P	64	Yes	Yes	Novel	LP
							76	No	NA		
c.3868C > G	15	p.Leu1290Val	p.Gln1653*	T	B	P	16	Yes	Yes	Novel	LB
c.3931G>A	15	p.Ala1311Thr		NT	B	P	80	No	No	Known	LB
c.4273C > T	15	p.Arg1425Cys	p.Gln3838*	NT	B	P	15	Yes	Yes	Novel	LB
c.5600A > G	15	p.Asn1867Ser	p.Arg400*	NT	PRD	D	17	Yes	Yes	Novel	USV
c.5957C>T	15	p.Thr1986Met		NT	PRD	P	87	Yes	NA	Novel	LB
c.6658C>T	15	p.Arg2220Trp		NT	PRD	D	85	Yes	Yes	Known	LP
c.6704C>T	15	p.Ser2235Leu		NT	PRD	D	70	Yes	Yes	Novel	LP
c.6827 T>C	15	p.Leu2276Pro		NT	PRD	D	61	Yes	Yes	Known	LP
c.6878C > T	15	p.Pro2293Leu	p.Pro36Leufs*78	NT	POD	P	12	Yes	NA	Known	LB
c.7099 T>C	17	p.Ser2367Pro		NT	PRD	D	56	Yes	NA	Novel	LP
c.7144A>C	17	p.Ser2382Arg		NT	PRD	D	67	Yes	Yes	Novel	LP
c.7241C > T	18	p.Thr2414Met	c.11269 + 1G > A	NT	PRD	D	10	Yes	Yes	Known	LP
c.7589G>A	19	p.Gly2530Asp		NT	PRD	D	59	No	NA	Known	LP
c.8158A>C	22	p.Thr2720Pro		NT	PRD	D	86	NA	NA	Novel	LP
c.8311G>A	23	p.Glu2771Lys		NT	PRD	D	83	Yes	NA	Known	LP
c.8744A > G	23	p.Asn2915Ser	p.Ser4190Serfs*21	T	B	D	28	Yes	Yes	Novel	USV
c.8750C > T	23	p.Ala2917Val	p.Gln3838*	T	POD	P	15	Yes	Yes	Known	LB
			p.Gln1653*				16	Yes	Yes		
c.10937 T>G	37	p.Val3646Gly		T	PRD	D	68	Yes	Yes	Novel	LP
c.10951G>A	37	p.Gly3651Ser		T	PRD	D	79	Yes	NA	Known	LP
c.11156G>T	38	p.Arg3719Leu		NT	PRD	D	82	Yes	Yes	Novel	LP
c.11248C>G	39	p.Arg3750Gly		NT	PRD	D	74	No	Yes	Known	LP
c.11257C>T	39	p.Arg3753Trp		NT	PRD	D	84	Yes	Yes	Known	LP
c.11351G > T	40	p.Gly3784Val		T	B	P	75	Yes	Yes	Novel	LB
c.11453G>A	41	p.Gly3818Asp		NT	PRD	D	81	Yes	Yes	Known	LP
c.11945A>C	43	p.Gln3982Pro		NT	PRD	P	78	Yes	Yes	Novel	LP
c.12671C>A	46	p.Thr4224Asn		NT	POD	P	66	Yes	NA	Novel	USV
*PKD2*											
c.965G>A	4	p.Arg322Gln		NT	PRD	D	64	Yes	NA	Known	LP
*GANAB*											
c.518G>A	5	p.Arg173Gln		T	PRD	D	58	Yes	NA	Novel	B
							86	NA	NA		
c.991C>T	10	p.Arg331Cys		NT	PRD	D	61	Yes	No	Novel	LB
c.1078A>G	11	p.Met360Val		NT	B	D	62	Yes	NA	Novel	USV
c.367C>G	4	p.Pro123Ala		T	POD	D	69	Yes	Yes	Novel	USV
c.2292A>G	19	p.Ile764Met		T	B	D			Yes	Novel	USV

*NT* Not Tolerated, *T* Tolerated, *PRD* Probably damaging, *B* Benign, *POD* Possibly damaging, *D* Disease causing, *P* Polymorphism, *LB* likely benign variation, *LP* likely pathogenic variation, *USV* uncertain significance variation, *NA* not available, *translation termination codon

### Evaluation of the pathogenicity of variations

We evaluated the potential pathogenicity of all identified missense variants according to the ACMG standards and guidelines for the interpretation of variations. The results are shown in Tables 1 and 2. A total of 84 variations were identified in *PKD1*, including 52 definitely pathogenic variations and 32 missense variants, 20 of which are classified as likely pathogenic mutations. Only four variants were identified in *PKD2*; three of them are definitely pathogenic mutations, and another is classified as likely to be pathogenic. Among the 76 definitely pathogenic or likely pathogenic mutations of *PKD1* and *PKD2*, 38 are novel. We identified five novel variations in *GANAB*. Two variations (p.Arg173Gln, and p.Arg331Cys) have been reported in the gnomAD database 431, and 1979 times, respectively, including 3, and 12 homozygotes, respectively, and are unlikely to be pathogenic. Three other variations (p.Pro123Ala, p.Met360Val, and p.Ile764Met) were identified in the gnomAD database 53,113, and 44 times, respectively, and are not very highly conserved. Two of these (p.Pro123Ala and p.Ile764Met) and a likely pathogenic mutation in PKD1 (p.Leu727Pro) were identified in family 69 with co-occurrence, and segregated with the disease in three affected family members. However, since functional analysis has not been performed, we are unable to determine their pathogenicity thus far, and they are classified as variants of uncertain significance.

### Correlation with male infertility

**Fig. 1:**
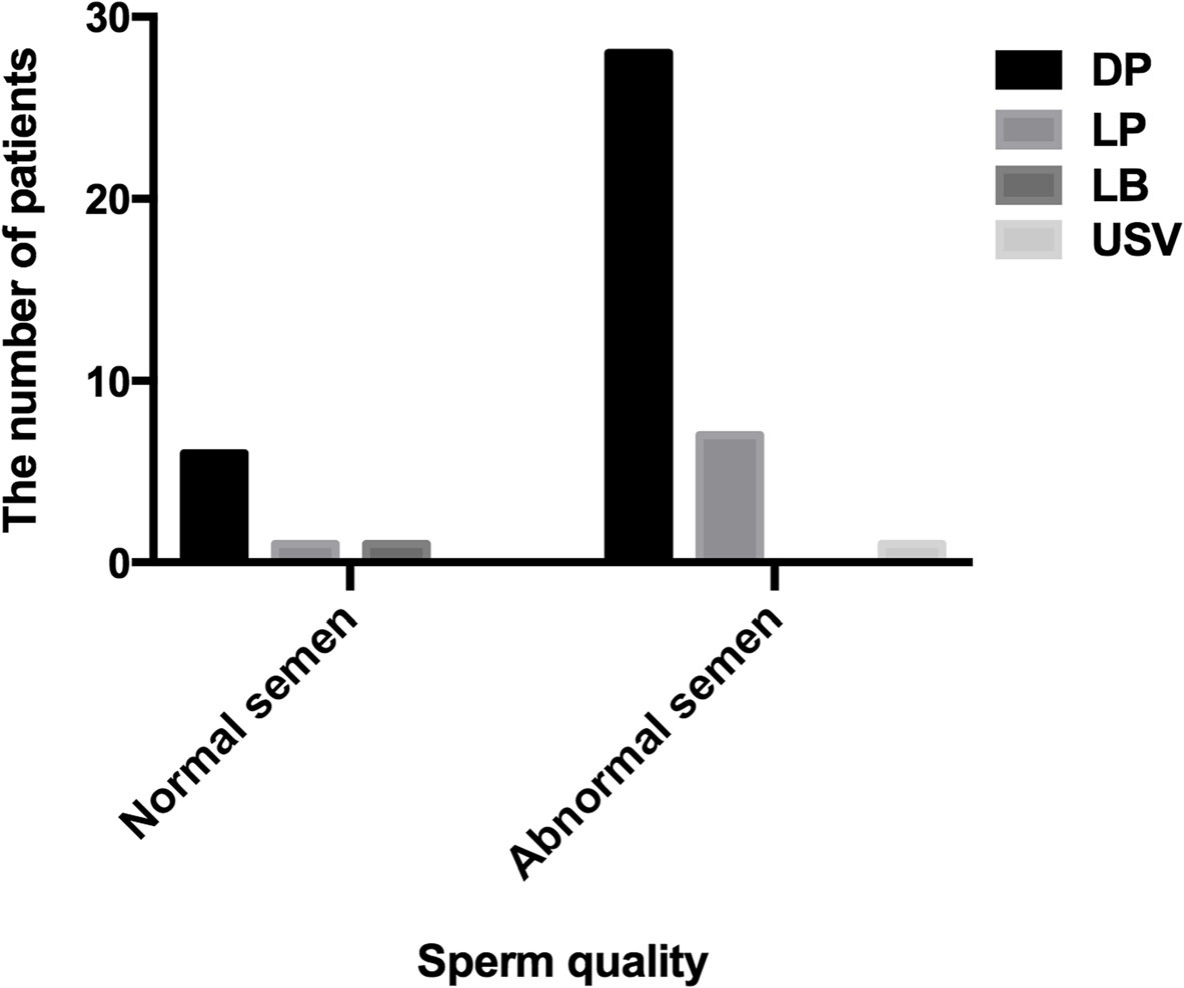
The semen quality of the male patients who harboured PKD1 mutations. DP, LP, LB and USV are indicated with definitely pathogenic mutations, likely pathogenic variations, likely benign variations and uncertain significance variations, respectively. The results showed that there is no correlation between semen quality and the type of mutation in *PKD1* gene

In order to analyze the correlation between ADPKD mutations and male infertility, we analyzed the types of PKD genes mutations and semen quality. In our study, the analysis of semen from 46 male patients revealed that sperm from 37 individuals were abnormal; asthenozoospermia was detected in 18 individuals; 18 other individuals were affected with oligozoospermia or oligoasthenozoospermia; and 1 individual suffered from azoospermia. A total of 28 of the individuals with abnormal sperm were found to harbour definitely pathogenic mutations, and 7 individuals with normal sperm also carried definitely pathogenic mutations (Table 3, Fig. 1). The results showed no correlation between semen quality and types of mutation in PKD genes.

**Table 3:** The semen analysis of 46 male patients

Gene	cDNA change	Exon/ intron	Amino acid change	Predicted location within PKD1 domains	Family No.	Age^a^	Inheriting/age^b^	Semen analysis
PKD1	c.856_862delTCTGGCC	5	p.Ser286Serfs*2	Ig-like repeat domain	30	27	maternal	oligoasthenozoospermia
PKD1	c.1385G>T	6	p.Arg462Met	C-type lectin domain	77	30	paternal/25	asthenozoospermia
PKD1	c.2527 T > C	11	p.Ser843Pro	not defined	45	34	paternal/22	oligoasthenozoospermia
PKD1	c.7126C > T	17	p.Gln2376*	REJ				
PKD1	c.2670 + 1G > A	IVS14	–	Ig-like repeat domain	19	34	maternal	asthenozoospermia
PKD1	c.3613G>C	15	p.Asp1205His	Ig-like repeat domain	64	30	paternal/28	asthenozoospermia
PKD2	c.965G>A	4	p.Arg322Gln	–				
PKD1	c.4447C > T	15	p.Gln1483*	Ig-like repeat domain	13	30	paternal/25	oligoasthenozoospermia
PKD1	c.4551C > A	15	p.Tyr1517*	Ig-like repeat domain	39	31	maternal	asthenozoospermia
PKD1	c.4609G > T	15	p.Glu1537*	Ig-like repeat domain	31	37	paternal/29	asthenozoospermia
PKD1	c.5957C>T	15	p.Thr1986Met	Ig-like repeat domain	87	38	paternal/23	asthenozoospermia
PKD1	c.6115C > T	15	p.Gln2039*	Ig-like repeat domain	55	36	paternal/25	asthenozoospermia
PKD1	c.6199C > T	15	p.Gln2067*	Ig-like repeat domain	34	23	maternal	oligoasthenozoospermia
PKD1	c.6658C>T	15	p.Arg2220Trp	REJ	85	31	paternal/22	oligoasthenozoospermia
PKD1	c.6704C>T	15	p.Ser2235Leu	REJ	70	30	paternal/29	asthenozoospermia
PKD1	c.7241C > T	18	p.Thr2414Met	REJ	10	30	maternal	oligoasthenozoospermia
PKD1	c.11269 + 1G > A	IVS39	–	not defined				
PKD1	c.7863 + 1G > C	IVS20	–	REJ	36	42	maternal	oligoasthenozoospermia
PKD1	c.7863 + 2 T > G	IVS20	–	REJ	11	33	maternal	asthenozoospermia
PKD1	c.7915C > T	21	p.Arg2639*	REJ	54	35	paternal/32	asthenozoospermia
PKD1	c.7973_7974delTG	21	p.Val2658Glyfs*2	REJ	9	33	paternal/28	asthenozoospermia
PKD1	c.8744A > G	23	p.Asn2915Ser	not defined	28	34	maternal	oligoasthenozoospermia
PKD1	c.12570_12571insCTCC	46	p.Ser4190Serfs*21	not defined				
PKD1	c.9666_9667delGA	28	p.Glu3222Aspfs*30	not defined	32	29	paternal/24	oligoasthenozoospermia
PKD1	EX31-33del	31–33	–	not defined	72	35	de novo	oligozoospermia
PKD1	c.10220 + 2 T > C	IVS32	–	not defined	3	44	NA	oligoasthenozoospermia
PKD1	c.12053C > T	44	p.Thr4018Ile	not defined				
PKD1	c.10397C > G	34	p.Ser3466*	not defined	6	27	paternal/22	oligoasthenozoospermia
PKD1	c.10524_10525delAG	35	p.Glu3509Aspfs*117	not defined	2	28	NA	oligoasthenozoospermia
PKD1	c.6804delG	15	p.Trp2268Cysfs*46	REJ	63	35	de novo	azoospermia
PKD1	c.10710_10715delGGCTGT	36	p.3571_3572del2	Putative TM region	40	34	maternal	asthenozoospermia
PKD1	c.10896_10897delGA	37	p.Ser3633Profs*88	not defined	5	37	de novo	oligoasthenozoospermia
PKD1	c.10937 T>G	37	p.Val3646Gly	not defined	68	32	paternal/26	asthenozoospermia
PKD1	c.10951G>A	37	p.Gly3651Ser	not defined	79	35	maternal	oligoasthenozoospermia
PKD1	c.11240delC	39	p.Pro3747Hisfs*79	not defined	33	33	paternal/25	oligoasthenozoospermia
PKD1	c.11538-2A > G	IVS41	–	not defined	18	36	paternal/30	oligoasthenozoospermia
PKD1	c.11617_11637del21	42	p.3873_3879del7	not defined	4	25	NA	asthenozoospermia
PKD1	c.11699_11700ins10	42	p.Leu3901Alafs*63	Putative TM region	22	29	paternal/22	asthenozoospermia
PKD1	c.11830_11838dup	43	p.Leu3944_Ala3946dup	Putative TM region	52	26	paternal/21	oligoasthenozoospermia
PKD1	c.11945A>C	43	p.Gln3982Pro	Putative TM region	78	33	paternal/28	asthenozoospermia
PKD1	c.12712C > T	46	p.Gln4238*	not defined	46	34	maternal	asthenozoospermia
PKD3	c.518G>A	5	p.Arg173Gln	–	58	37	paternal/27	asthenozoospermia
PKD1	c.1198C > T	5	p.Arg400*	not defined	17	29	maternal	normal
PKD1	c.5600A > G	15	p.Asn1867Ser	Ig-like repeat domain				
PKD1	c.3931G>A	15	p.Ala1311Thr	Ig-like repeat domain	80	27	de novo	normal
PKD1	c.2039A > T	10	p.Tyr680Phe	not defined	20	27	paternal/27	normal
PKD1	c.5637C > G	15	p.Tyr1879*	Ig-like repeat domain				
PKD1	c.4273C > T	15	p.Arg1425Cys	Ig-like repeat domain	15	31	paternal/28	normal
PKD1	c.8750C > T	23	p.Ala2917Val	not defined				
PKD1	c.11512C > T	41	p.Gln3838*	Putative TM region				
PKD1	c.6813_6814delAC	15	p.Arg2272Glyfs*147	REJ	7	32	NA	normal
PKD1	c.7144A>C	17	p.Ser2382Arg	REJ	67	29	maternal	normal
PKD1	c.10050 + 1G > A	IVS30	–	not defined	44	40	paternal/30	normal
PKD1	c.12139-2A > T	IVS44	–	Putative TM region	24	34	maternal	normal
PKD2	c.2159dupA	11	p.Asn720Lysfs*5	–	42	29	maternal	normal

*REJ* receptor for egg jelly; *NA* not available; ^a^the age of the male patients seeking fertility advice from doctors; ^b^the age of the patients’ fathers fathering their last child; *translation termination codon

A total of 35 patients who provided semen specimens for analysis have chosen different approaches to conceive. Two patients (one with asthenozoospermia and the other with oligoasthenozoospermia) conceived naturally, and two individuals (one with asthenozoospermia and the other with normal sperm) conceived through ICSI. The 31 other patients chose ICSI combined with PGD; of these patients, genetic diagnosis of embryos has been completed for 22 and the treatment cycle is currently incomplete for 9 patients (Table 4).

**Table 4:** The assisted reproductive therapies used by the 35 male patients and the clinical outcomes of those therapies

Gene	cDNA change	Amino acid change	Family No.	Semen analysis	Treatment methods
PKD1	c.856_862delTCTGGCC	p.Ser286Serfs^*^2	30	oligoasthenozoospermia	ICSI+PGD
PKD1	c.1385G>T	p.Arg462Met	77	asthenozoospermia	ICSI+PGD
PKD1	c.2527 T > C	p.Ser843Pro	45	oligoasthenozoospermia	ICSI+PGD
PKD1	c.7126C > T	p.Gln2376^*^			
PKD1	c.2670 + 1G > A	–	19	asthenozoospermia	ICSI+PGD^a^
PKD1	c.4447C > T	p.Gln1483^*^	13	oligoasthenozoospermia	ICSI+PGD^a^
PKD1	c.4551C > A	p.Tyr1517^*^	39	asthenozoospermia	natural pregnant
PKD1	c.4609G > T	p.Glu1537^*^	31	asthenozoospermia	ICSI+PGD
PKD1	c.6199C > T	p.Gln2067^*^	34	oligoasthenozoospermia	natural pregnant
PKD1	c.6658C>T	p.Arg2220Trp	85	oligoasthenozoospermia	ICSI+PGD
PKD1	c.6704C>T	p.Ser2235Leu	70	asthenozoospermia	ICSI+PGD
PKD1	c.7863 + 1G > C	–	36	oligoasthenozoospermia	ICSI+PGD
PKD1	c.7863 + 2 T > G	–	11	asthenozoospermia	ICSI+PGD
PKD1	c.10529C > T	p.Thr3510Met			
PKD1	c.7915C > T	p.Arg2639^*^	54	asthenozoospermia	ICSI+PGD
PKD1	c.7973_7974delTG	p.Val2658Glyfs^*^2	9	asthenozoospermia	ICSI
PKD1	c.10529C > T	p.Thr3510Met			
PKD1	c.8744A > G	p.Asn2915Ser	28	oligoasthenozoospermia	ICSI+PGD^a^
PKD1	c.12570_12571insCTCC	p.Ser4190Serfs^*^21			
PKD1	c.9666_9667delGA	p.Glu3222Aspfs^*^30	32	oligoasthenozoospermia	ICSI+PGD^a^
PKD1	c.10220 + 2 T > C	–	3	oligoasthenozoospermia	ICSI+PGD^a^
PKD1	c.12053C > T	p.Thr4018Ile			
PKD1	c.10524_10525delAG	p.Glu3509Aspfs*117	2	oligoasthenozoospermia	ICSI+PGD
PKD1	c.10896_10897delGA	p.Ser3633Profs*88	5	oligoasthenozoospermia	ICSI+PGD^a^
PKD1	c.10937 T>G	p.Val3646Gly	68	asthenozoospermia	ICSI+PGD
PKD1	c.11240delC	p.Pro3747Hisfs^*^79	33	oligoasthenozoospermia	ICSI+PGD^a^
PKD1	c.11538-2A > G	–	18	oligoasthenozoospermia	ICSI+PGD
PKD1	c.11699_11700ins10	p.Leu3901Alafs^*^63	22	asthenozoospermia	ICSI+PGD
PKD1	c.11830_11838dup	p.Leu3944_Ala3946dup	52	oligoasthenozoospermia	ICSI+PGD
PKD1	c.11945A>C	p.Gln3982Pro	78	asthenozoospermia	ICSI+PGD
PKD1	c.12712C > T	p.Gln4238^*^	46	asthenozoospermia	ICSI+PGD
PKD1	c.1198C > T	p.Arg400^*^	17	normal	ICSI+PGD
PKD1	c.5600A > G	p.Asn1867Ser			
PKD1	c.3931G>A	p.Ala1311Thr	80	normal	ICSI
PKD1	c.2039A > T	p.Tyr680Phe	20	normal	ICSI+PGD^a^
PKD1	c.5637C > G	p.Tyr1879^*^			
PKD1	c.4273C > T	p.Arg1425Cys	15	normal	ICSI+PGD^a^
PKD1	c.8750C > T	p.Ala2917Val			
PKD1	c.11512C > T	p.Gln3838^*^			
PKD1	c.6813_6814delAC	p.Arg2272Glyfs^*^147	7	normal	ICSI+PGD
PKD1	c.7144A>C	p.Ser2382Arg	67	normal	ICSI+PGD
PKD1	c.10050 + 1G > A	–	44	normal	ICSI+PGD
PKD1	c.12139-2A > T	–	24	normal	ICSI+PGD
PKD2	c.2159dupA	p.Asn720Lysfs^*^5	42	normal	ICSI+PGD

*ICSI* intracytoplasmic sperm injection, *ICSI+PGD* ICSI combined preimplantation genetic diagnosis, ^a^the treatment cycle is currently incomplete, *translation termination codon

## Discussion

In the present study, we analysed 90 unrelated patients affected with polycystic kidney disease, including 37 male patients with infertility. Screening of *PKD1*, *PKD2,* and *GANAB* was performed using a series of molecular genetic analyses. A total of 76 mutations (definitely or likely pathogenic mutations) were identified in 77 of the families, comprising 72 mutations in *PKD1* and 4 in *PKD2*. Pathogenic mutations in *GANAB* have never been identified in Chinese patients. To our knowledge, this is the first report of *GANAB* screening in a cohort of Chinese patients with ADPKD.

*PKD1*, *PKD2,* and *GANAB* are located in chromosome regions 16p13.3, 4q21–22, and 11q12.3, and they produce the proteins polycystin-1 (PC-1), PC-2, and neutral alpha-glucosidase AB, respectively [17]. A series of molecular genetic analyses were used to screen for mutations of PKD genes. The human genome contains six truncated *PKD1* pseudogenes, which share approximately 97.7% similarity with exons 1–34 of *PKD1* [18]. *PKD1* contains complex reiterated regions, necessitating that LR-PCR be performed prior to sequencing [19]. Screening of *PKD2* was performed by direct Sanger sequencing. Subsequently, MLPA was employed to analyse the copy number variations of *PKD1* and *PKD2* in the genetically unresolved families, followed by screening of *GANAB* using direct Sanger sequencing. This strategy can typically identify almost all variants in PKD genes, as verified by our high mutation detection rate (85.6%,77/90). Recently, targeted next-generation sequencing and whole-exome sequencing have been used to identify mutations involved in ADPKD. Although these methods have high sensitivity, specificity, and accuracy, LR-PCR is still required; furthermore, highly specialised personnel and expensive equipment are required [20]. Thus, the strategy for the identification of mutations in ADPKD used in the present study may be useful in a wide variety of situations.

A total of 2609 variants had been described before January 2018 (2323 in *PKD1*, 278 in *PKD2*, and 8 in *GANAB*). The majority of these variants were missense variants (1225). The others were protein-truncating variants (840), splice site mutations (165), in-frame indels (115), large deletions (24), and variations in the UTR and intervening sequences (228). In our study, a total of 76 mutations (definitely or likely pathogenic variations) were identified in 77 of the families, comprising 41 protein-truncating, 21 missense mutations, and 9 splice site mutations; 4 in-frame indels; and 1 large deletion variants. The positive detection rate was 85.6% (77/90), and 50% (38/76) of all mutations were novel. The proportion of patients with ADPKD with a family history of the disease accounted for 80% (72/90) of all probands, comparable to previously published data [21].

A total of 76 mutations were identified, 72 of which (including 52 definitely pathogenic mutations) were in *PKD1,* accounting for 94.7%. Among all definitely pathogenic mutations of *PKD1*, 39 were truncating mutations, accounting for a large proportion (75%, 39/52), concordant with the results of other recent studies [17]. Furthermore, one large deletion of *PKD1* was identified in our set of patients (1.1%, 1/90), in accordance with previously reported results [22, 23]. A total of 4 mutations were identified in *PKD2*, 3 of which were definitely pathogenic mutations. However, no hot-spots of mutation were identified in *PKD1* or *PKD2*, indicating that for identification of future mutations, all exons of *PKD1* and *PKD2*, including their intron-exon boundaries, should be sequenced.

*GANAB* has been implicated in the development of autosomal-dominant polycystic kidney and liver disease [7]. In this study, a total of five mutations with a frequency below 1% were identified in the Asian population of the Exac and gnomAD databases; all are missense mutations. Only two mutations (p.Pro123Ala and p.Ile764Met) co-occurred in family 69; these have been described a few times and were found to have segregated with the disease in affected family members, accounting for 1.1%. The patients in this family all suffered from polycystic kidney with liver disease, consistent with earlier findings that the phenotype caused by *GANAB* mutations usually manifests with polycystic liver disease (PLD) [7]. In addition, the patients from family 69 also carried the p.Leu727Pro mutation in *PKD1*, which has been reported in several families and classified as a highly likely pathogenic mutation [17, 24]. As p.Pro123Ala was predicted to be a disease-causing mutation by three tools and p.Ile764Met was predicted to be benign by PolyPhen-2, the possibility that p.Leu727Pro in *PKD1* and p.Pro123Ala in *GANAB* co-contribute to the development of polycystic kidney with liver disease cannot be excluded. Thus, the fact that no definitely or likely pathogenic mutation was detected in this study suggested that GANAB mutations are rare in Chinese patients with ADPKD.

Earlier studies have reported that *HNF1B* can phenocopy ADPKD [25–27]. In addition, it has been very recently reported that monoallelic mutation in *DNAJB11* can cause atypical ADPKD, which is a phenotypic hybrid of ADPKD and autosomal-dominant tubulointerstitial diseases (ADTKD) [28]. In our study, for the 13 patients with or without a positive family history, the genetic cause remains unknown, but undetected *PKD1*, *PKD2,* or *GANAB* mutations, including deep intronic or synonymous exonic mutations that cause atypical splicing, or large deletions of *GANAB*, could be the underlying reasons. Furthermore, the patients should be reevaluated based on their most recent phenotype, and should be screened for other genes implicated in ADPKD for future analysis, such as *HNF1B* and *DNAJB11*.

ADPKD is a systemic disorder and extrarenal manifestation is not uncommon. Male patients with ADPKD usually suffer from infertility, resulting from abnormal semen, including necrospermia, immotile sperm, asthenozoospermia, and azoospermia [29–31]. PKD1 and PKD2 have been reported to play a pivotal role in the development and maintenance of the male reproductive tract [32, 33]. The potential aetiologies of semen abnormalities in male patients with ADPKD include ejaculatory duct cysts, seminal vesicle cysts, and ultrastructural flagellar defects caused by abnormal polycystins [30]. However, the correlation between the type of PKD gene mutation and semen quality remains unclear. In the present study, 37 individuals were found to have abnormal semen (80%, 37/46). Only some of the male patients with ADPKD carrying definitely pathogenic mutations were infertile, which may indicate that there is no correlation between the type of PKD1 mutation and semen quality. However, 23 of the variations are located in the same four domains of *PKD1*, and more than one third of the mutations (39%,9/23) are located in the Ig-like repeat domain, which is a conserved region of approximately 85 bp surrounding a central sequence consisting of 16 copies [34]. Defects of the Ig-like repeat domain in PKD1 protein may alter its binding ability, leading to male reproductive tract cysts and infertility [34]. Thus, male patients carrying pathogenic mutations in *PKD1* located in the Ig-like repeat domain may have a high risk of infertility. In addition, 20 and 3 of the mutations identified in the 23 male patients with ADPKD with abnormal semen were paternal and de novo, respectively. However, almost all of these fathers fathered their children when they were younger than the age at which their sons with ADPKD sought fertility advice from their doctors. Furthermore, after the treatment of ICSI, 17 individuals affected with semen abnormalities all achieved paternity or at least obtained embryos. Therefore, we suggest that male patients with ADPKD should achieve paternity as young as possible, and the use of ICSI combined PGD should be considered for patients suffering from low semen quality [30, 31].

In this study, the majority of male patients with ADPKD were found to have abnormal semen (80%), which could due to a selection bias in the study population. Since our hospital specializes in reproductive and genetic disorders, most of the subjects included in the study visited our hospital to seek treatment for infertility. Thus, the proportion of males with abnormal sperm quality is not a true reflection of the proportion of male ADPKD patients with abnormal sperm. Therefore, studies on larger groups of patients with ADPKD recruited from general hospitals are needed to obtain a more accurate estimation of the proportion of ADPKD-affected males with abnormal sperm.

## Conclusions

In conclusion, we identified a group of novel mutations in PKD genes, which enriches the PKD mutation spectrum. Male patients with ADPKD are usually affected with infertility, and surgical sperm retrieval combined with assisted reproductive technology may help them to achieve paternity. Our study will provide clinicians with precise diagnoses that have implications for family planning and genetic counselling of affected individuals.

## Additional files

Additional file 1: **Table S1.** Primers used in long-range PCR of *PKD1* homologous regions. (DOC 37 kb)

Additional file 2: **Table S2.** Primers used in nested PCR of *PKD1* homologous regions. (DOC 78 kb)

Additional file 3: **Table S3.** Primers used in PCR of *PKD1* single-copy regions. (DOC 40 kb)

Additional file 4: **Table S4.** Primers used in PCR of *PKD2* and *GANAB*. (DOC 53 kb)

## Abbreviations

ACMG: American College of Medical Genetics and Genomics
ADPKD: Autosomal dominant polycystic kidney disease
ESRD: End-stage renal disease
HGMD: Human gene mutation database
ICSI: Intracytoplasmic sperm injection
LR-PCR: Long-range PCR
MLPA: Multiplex ligation-dependent probe amplification
PC-1: Produce the proteins polycystin-1
PC-2: Produce the proteins polycystin-2
PKDB: Autosomal dominant polycystic kidney disease mutation database
PLD: Polycystic liver disease
WHO: World Health Organization
