# Supplementary material for: Novel mutations of PKD genes in Chinese patients suffering from autosomal dominant polycystic kidney disease and seeking assisted reproduction
Source: BMC Med Genet. 2018 Oct 17;19:186. doi: 10.1186/s12881-018-0693-7 (PMC6192368; doi:10.1186/s12881-018-0693-7)
Supplement: Supplementary file 1 — Table S1. Primers used in long-range PCR of PKD1 homologous regions. (DOC 37 kb) [file 12881_2018_693_MOESM1_ESM.doc]

**Supplementary table1 Primers used in long-range PCR of *PKD1* homologous regions**

| Primers | Exons | Primer sequence 5’-3’ | | TM（℃） |
| --- | --- | --- | --- | --- |
|  |  | Forward sequence | Reverse sequence |  |
| PKD1-L1 | 1 | CCATCCACCTGCTGTGTGACCTGGTAAAT | CCACCTCATCGCCCCTTCCTAAGCAT | 69 |
| PKD1-L2 | 2-7 | ATTTTTTGAGATGGAGCTTCACTCTTGCAGG | CGCTCGGCAGGCCCCTAACC | 69 |
| PKD1-L3 | 8-12 | CCGCCCCCAGGAGCCTAGACG | CATCCTGTTCATCCGCTCCACGGTTAC | 69 |
| PKD1-L4 | 13-15 | TGGAGGGAGGGACGCCAATC | GTCAACGTGGGCCTCCAAGT | 69 |
| PKD1-L5 | 15-21 | AGCGCAACTACTTGGAGGCCC | GCAGGGTGAGCAGGTGGGGCCATCCTA | 69 |
| PKD1-L6 | 22 | GAGGCTGTGGGGGTCCAGTCAAGTGG | AGGGAGGCAGAGGAAAGGGCCGAAC | 72 |
| PKD1-L7 | 23-28 | CCCCGTCCTCCCCGTCCTTTTGTC | AAGCGCAAAAGGGCTGCGTCG | 69 |
| PKD1-L8 | 29-34 | GGCCCTCCCTGCCTTCTAGGCG | GTTGCAGCCAAGCCCATGTTA | 69 |
