# Supplementary material for: Novel mutations of PKD genes in Chinese patients suffering from autosomal dominant polycystic kidney disease and seeking assisted reproduction
Source: BMC Med Genet. 2018 Oct 17;19:186. doi: 10.1186/s12881-018-0693-7 (PMC6192368; doi:10.1186/s12881-018-0693-7)
Supplement: Supplementary file 2 — Table S2. Primers used in nested PCR of PKD1 homologous regions. (DOC 78 kb) [file 12881_2018_693_MOESM2_ESM.doc]

**Supplementary table2 Primers used in nested PCR of *PKD1* homologous regions**

| Primers | Primer sequence 5’-3’ | | TM（℃） |
| --- | --- | --- | --- |
|  | Forward sequence | Reverse sequence |  |
| PKD1-1 | TGCGAGCCCCCCTGCCTC | AACCCGCCCACGCCCGCCCGTCC | 70 |
| PKD1-2/3 | TAGGGGCTCTGGCCCTGAC | CCAGCCAGGACCCCACCCAAAG | 62 |
| PKD1-4 | CATAGACCCTTCCCACCAG | CCTGGCTGGGAAGGACAGA | 61 |
| PKD1-5a | TGGAGCCAGGAGGAGCAGAA | CAGAGGGACAGGCAGGCAAA | 62 |
| PKD1-5b | AGCCCTCCAGTGCCTCCTTT | GCACGGCCGTCACGTGATAG | 64 |
| PKD1-5c | TGGGACTTCGGAGACGGCT | GAGTGGGCAGCAGACACTCA | 63 |
| PKD1-6 | ACCGTTGACACCCTCGTTCC | TCTCTGCCCCAGTGCTTCAG | 62 |
| PKD1-7 | CTAACCACAGCCAGCGTCTC | CTGTGAGGGTGGGAGGATGG | 62 |
| PKD1-8 | GCGGCTCGGTCCCCAGTCT | GGAGGGCAGGTTGTAGAACGTG | 64 |
| PKD1-9 | GGAGTCTGGGCTTCAGGCTG | CACCCACCACCCAGAGTCCC | 62 |
| PKD1-10a | GGCCTGTGGGCAAATCAGGG | TGGGGGTGGCAGGAGGCGTC | 62 |
| PKD1-10b | AGGGGGACGCTGGTGCCCTG | GGGAACAGACCCAGGTCAGG | 61 |
| PKD1-11a | GTCCACGGGCCATGACCG | CCAGCCACTGGGGAGACCAC | 63 |
| PKD1-11b | GGCAGAGGTGGGCAATGG | AGCCGGGCACGAAGGTGGC | 62 |
| PKD1-11c | GTGTCAGCGCCCGCTTTG | CTGTGTGAGCACCCTGTCTGC | 62 |
| PKD1-12 | CTCCACAGAGCCCAGGCTG | ATCCCCTCCCCTCCCCACCC | 61 |
| PKD1-13 | CTGCCACCTGGGCTCACTG | TGCCCACCCCAAACCGGC | 60 |
| PKD1-14 | CTCACTGCGTCCCACCGC | CTGAAAGGCAGTGGCCCC | 60 |
| PKD1-15a | TGGGGAGCAGGTGGGGGTGC | AGACGCGCACATCCGCCTGGGCCG | 63 |
| PKD1-15b | CGTGCGCCTGGAGGTCAAC | GGCTGCGTGGGGATGCAG | 63 |
| PKD1-15c | CGTGCTGGTCTTCGTCCTGG | TGTAGCGGTAGGGGAACGG | 61 |
| PKD1-15d | GTTTGTGCAGCTCGGGGAC | AAGCGTGGGTGACCTCCG | 61 |
| PKD1-15e | CCCGCCAGCTACCTGTGG | GCGGAGCCCACCTCGTTC | 61 |
| PKD1-15f | CTTCCGCTCCGTGGGCAC | GGAGGCGGCCACCATCAG | 61 |
| PKD1-15g | AGCGCCTGGGCCGACTGCAC | AGCTGCCCCCAAAAGGGC | 63 |
| PKD1-15h | GAGCCCGGAGGCAGCTTC | GGGAGCACCTCGGGGTTG | 63 |
| PKD1-15i | AGCTGTCACCTTCCGCCTG | GCACCTGGATCTCCAACAGCC | 62 |
| PKD1-15l | GCTGGTCATCCTGTCGGGC | CACCAGGTTGGAGGCGTTC | 62 |
| PKD1-15m | CCAGGGCCGAGCACTCCTAC | GTCAACGTGGGCCTCCAAGT | 60 |
| PKD1-15n | AGCGCAACTACTTGGAGGCCC | TGGGGTCGTAGGACTCGCTC | 62 |
| PKD1-15p | CGCCTGGTGCCCATCATTG | GGACGGGTGAGGGGCATG | 60 |
| PKD1-16 | AGGCCACGTCGCCCCTTG | GAGGCTGGGCTGTCCAAGG | 63 |
| PKD1-17 | GAGGTAACCCCACTCCCACG | ATCCCCAGCCCGCCCACAC | 60 |
| PKD1-18 | AGAGGGTTGCGCCCCCTC | ATCCCGCTGCTCCCCCCACGCAGG | 63 |
| PKD1-19 | TCCCGTGATGCCGTGGGG | CAGGTGGCAGTCTCGGGG | 62 |
| PKD1-20 | CCACCTGCTCACCACCCC | GCAGGGGTACAGGTCTTGGTCCC | 62 |
| PKD1-21 | GCGCTGCTGACAGCTTGC | ATGCGGGGCAGGGTGAGC | 60 |
| PKD1-22 | AGTGGGGCCAGGAGCGGG | GGGCGGGTGGCATGGGGC | 62 |
| PKD1-23a | CCCTCCCTCTACCTCCCTGTC | CACTGAGGTTGGCCAGGGC | 62 |
| PKD1-23b | GGGCCTGGCTGCCACTTC | AAGGCCAGGGGGCCGCGTG | 62 |
| PKD1-24 | CAGGCGTGTGACCTGCGC | TGCCCTGCCCTGCCAGCTG | 61 |
| PKD1-25 | CTGGGCTCACGTCCGCTAC | GCTCCCAGGAGCACAGGGTC | 61 |
| PKD1-26 | GAGAAGGCACAGCTTGCACG | AGAGCAGGGGAGGCCCTG | 62 |
| PKD1-27 | GCAGACCGAGCCTCCCAC | AGGGGCAGAGCTTGGCAG | 62 |
| PKD1-28 | TGCGAGCCTGACCTCCCTC | CCAACCTCCCACGGAGTGG | 61 |
| PKD1-29 | TTGGGCAGGGTGGTCCTG | GGAAGGGCTGGGCAGGAAG | 62 |
| PKD1-30 | CAGCCTCACCTGTGTGGCC | TCCATTCCCAGTACTCCCGG | 61 |
| PKD1-31/32 | GAGCAGGTCTGAGCTGCCG | GCACCAGGGCTCGAGGTTTC | 62 |
| PKD1-33 | GGGTGGGCTGTGTGTGTGAC | GCAAGGGTGAGCTTCAGAGCC | 62 |
| PKD1-34 | GCCCACCCTATGCCTCCTG | AATCCCCCCTCCCCCGAGAGCCGG | 60 |
