# Supplementary material for: Novel mutations of PKD genes in Chinese patients suffering from autosomal dominant polycystic kidney disease and seeking assisted reproduction
Source: BMC Med Genet. 2018 Oct 17;19:186. doi: 10.1186/s12881-018-0693-7 (PMC6192368; doi:10.1186/s12881-018-0693-7)
Supplement: Supplementary file 3 — Table S3. Primers used in PCR of PKD1 single-copy regions. (DOC 40 kb) [file 12881_2018_693_MOESM3_ESM.doc]

Supplementary table 3 Primers used in PCR of *PKD1* single-copy regions

| Primers | Primer sequence 5’-3’ | | TM（℃） |
| --- | --- | --- | --- |
|  | Forward sequence | Reverse sequence |  |
| PKD1-35 | AGGTTAACATGGGCTTGGCT | ACAGACCTGTGAGAGGCAGC | 60 |
| PKD1-36 | CAGGTCTGTCTCTGCTTCCC | AGGCCTGTAGCCTACCCCT | 60 |
| PKD1-37 | CTCTGAAGCCACCCCCTC | GTGGGAGACAAGAGACGGAG | 60 |
| PKD1-38 | CAAAGCCCTGCTGTCACTGT | AAAGCCCAGAAGACAGACCA | 60 |
| PKD1-39 | ATGTTCCCTGGGTCTCTGGT | ACGATTTAAGTCTTGGGGCA | 60 |
| PKD1-40 | CCTGTTGGGTTTTGATGAGG | CGGCACTCCTGGAGAACTAC | 60 |
| PKD1-41 | GTAGTTCTCCAGGAGTGCCG | GCTCCTGGCTGGTGACTG | 60 |
| PKD1-42 | TGCCACCCGCTCCTACTGA | TGGAGGCGCGGGGTCT | 60 |
| PKD1-43 | CGTCCCTCCCGCCCTCCTGA | TCTGTCTGCTTGCAGCCCTGGGGTGTG | 60 |
| PKD1-44 | GGCTGCAAGCAGACAGATTT | AGTGAGGGCGTACAGCTGAG | 60 |
| PKD1-45 | TCACTGGTGTCGCCTTCC | CACAGGGGCTCAGTCAGTC | 60 |
| PKD1-46.4 | AGAACAAGGTCCACCCCAG | TGAGGACTCGGGGAAATAAA | 60 |
| PKD1-46.5 | GGACAAGGTGTGAGCCTGAG | GCCAGGAAGGAGGACTAAGTG | 60 |
| PKD1-46.6 | GGACTGACTGAGCCCCTGT | AGTcGGTCAAACTGGGTGAG | 60 |
