# Supplementary material for: Novel mutations of PKD genes in Chinese patients suffering from autosomal dominant polycystic kidney disease and seeking assisted reproduction
Source: BMC Med Genet. 2018 Oct 17;19:186. doi: 10.1186/s12881-018-0693-7 (PMC6192368; doi:10.1186/s12881-018-0693-7)
Supplement: Supplementary file 4 — Table S4. Primers used in PCR of PKD2 and GANAB. (DOC 53 kb) [file 12881_2018_693_MOESM4_ESM.doc]

Supplementary table 4 Primers used in PCR of *PKD2* and *GANAB*

| Primers | Primer sequence 5’-3’ | | TM（℃） |
| --- | --- | --- | --- |
|  | Forward sequence | Reverse sequence |  |
| PKD2-e1 | CGCGCCGGACGCCAGTGACC | CCGGCCGTTCTGGTTCGTGCATC | 60 |
| PKD2-e2 | AAATGATATCTTTTCTTTTCTTCA | AACTTTCCCATTAGTGCAAG | 60 |
| PKD2-e3 | CCAAAATGTTTATCCACAGG | AGGTACTTTCAAAGTTATTTGCA | 60 |
| PKD2-e4 | TGGTTATGCAAACGATGCAGG | CCGAGTGGCAATGAGTCACA | 60 |
| PKD2-e5 | GCCTCAAGTGTTCCACTGAT | AGGTTTTTCTGGGTAACCCCAG | 60 |
| PKD2-e6 | TTTAATTGTTCTTATTTACATGCA | TTTAATTGTTCTTATTTACATGCA | 60 |
| PKD2-e7 | TTGGTGAAGAAAAATATACTAGTCA | TGGAACTCATTTTTTTTAAAGA | 60 |
| PKD2-e8 | TTTTATTATATACAGTCACACCA | CAACTCTGATTAAATTTTTCTTCTT | 60 |
| PKD2-e9 | TTTGGTTTTGTATTGTGGTG | AAGGATTTACGAAGTTTAAATTG | 60 |
| PKD2-e10 | TAATTCCAAATTATGTTTCTTCC | TGAAACAATGCTCATTTTATG | 60 |
| PKD2-e11 | AAACCAAGTCTTTTATTTTTTCTC | GGGCTAGAAATACTCTTATCACC | 60 |
| PKD2-e12 | GGAATGATTTTTATCTGTATCCTCTC | TAGGTACCAAATCAAATCCG | 60 |
| PKD2-e13 | GTCTCAGTGTTCTGCTCCTC | AAATTCTGCCAATTCCTTTA | 60 |
| PKD2-e14 | TGTACTGTGTTTTCCTTGCA | AAATACAACTGTCAGCAACATA | 60 |
| PKD2-e15 | ACACCAGTTTCTTTTTCCCT | ATCGGTCACAAAGACTAGCA | 60 |
| PKD3-1 | CGTGCAAAGTCTGACAGCG | GGCTTTACTAAACCGCCGGG | 58 |
| PKD3-2/3 | AGTGAGCCAACTTCTCAGGC | TCTAGCACCAGCAACACCTG | 58 |
| PKD3-4 | CTGATTCCCTCACGGTCCAT | ATGGCAAGGTGCTATGCTAGT | 58 |
| PKD3-5 | TAACAGGTAGATTTCCCCCGGTC | CACCCGGGTCTGGTTCCTA | 58 |
| PKD3-6 | CCTGGGTCTGTGTCTCCTTC | GACATCACACAGCAGGGGAA | 58 |
| PKD3-7/8/9 | TCCTCCAATGCCTTCGTTCC | TCCCTAAAGGCCTGACTCCT | 58 |
| PKD3-10 | CTCTAGGCAAGCATGGGTCA | GTGAGTGAGCGTGTCACCTG | 58 |
| PKD3-11 | CAGCTCTGTGTGGACTCTGG | AGGCAGGCTGAGGGGTATAA | 58 |
| PKD3-12/13 | TTATACCCCTCAGCCTGCCT | CCTAAGGGGCAAAAGAGCCA | 58 |
| PKD3-14/15/16 | CTGACTATGAGGGCTGGTGC | GCTCCTGAGATCTGGCCTTC | 58 |
| PKD3-17/18 | GGGAGGTCCACTCCTTGTTC | CCCAGTCCCTAATACCCCCA | 58 |
| PKD3-19/20/21 | TGGAGGAGCATTTCTGACTTGTG | CTGGACCCTAAGGCACACATT | 58 |
| PKD3-22/23 | GGGAATGTGTGCCTTAGGGTC | ATCACCACCCGCTCAATCC | 58 |
| PKD3-24/25 | GCTTTGGGTCTCCTCCTTCC | GAATTTGGAGCCCAGGGTCA | 58 |
